# Nationwide Initiation of Cardiovascular Risk Treatments During the COVID-19 Pandemic in France: Women on a Slippery Slope?

**DOI:** 10.3389/fcvm.2022.856689

**Published:** 2022-04-25

**Authors:** Amélie Gabet, Clémence Grave, Philippe Tuppin, Thomas Lesuffleur, Charles Guenancia, Viêt Nguyen-Thanh, Romain Guignard, Jacques Blacher, Valérie Olié

**Affiliations:** ^1^Santé Publique France, Paris, France; ^2^Caisse Nationale de l’Assurance Maladie, Paris, France; ^3^Department of Cardiology, University Hospital, Dijon, France; ^4^Centre de Diagnostic et de Thérapeutique, Hôtel-Dieu, AP-HP, Université de Paris, Paris, France

**Keywords:** cardiovascular, medication, initiation, cardiovascular risk, COVID-19 pandemic

## Abstract

**Objectives:**

This study examines the initiation of prescribed medication treatments for cardiovascular risk (antihypertensives, lipid-lowering drugs, oral anticoagulants in atrial fibrillation, and smoking cessation medications) during the COVID-19 pandemic in the French population.

**Methods:**

For each year between 2017 and 2021, we used the French National Insurance Database to identify the number of people with at least one reimbursement for these medications but no reimbursement in the previous 12 months. We computed incidence rate ratios (IRRs) between 2017–2019 and, respectively 2020 and 2021 using Poisson regression adjusted for age and 2017–2019 time trends. We recorded the number of lipid profile blood tests, Holter electrocardiograms, and consultations with family physicians or cardiologists.

**Results:**

In 2020, IRR significantly decreased for initiations of antihypertensives (−11.1%[CI95%, −11.4%;−10.8%]), lipid-lowering drugs (−5.2%[CI95%, −5.5%;−4.8%]), oral anticoagulants in atrial fibrillation (−8.6%[CI95%, −9.1%;−8.0%]), and smoking cessation medications (−50.9%[CI95%, −51.1%;−50.7%]) compared to 2017–2019. Larger decreases were found in women compared to men except for smoking cessation medications, with the sex difference increasing with age. Similar analyses comparing 2021 to 2017–2019 showed an increase in the initiation of lipid-lowering drugs (+ 11.6%[CI95%, 10.7%;12.5%]) but even lower rates for the other medications, particularly in women. In addition, the 2020 number of people visiting a family physician or cardiologist decreased by 8.4 and 7.4%. A higher decrease in these visits was observed in those over 65 years of age compared to those under 65 years of age. A greater use of teleconsultation was found in women.

**Conclusion:**

The COVID-19 pandemic heavily impacted the initiation of medication treatments for cardiovascular risk in France, particularly in women and people over 65 years.

## Introduction

Healthcare use has been challenged during the COVID-19 pandemic (1), particularly during the first epidemic wave and the first lockdown, which lasted from 17 March to 10 May 2020 in France (2, 3). Two subsequent lockdowns, although less restrictive than the first one, were implemented in France to contain the second epidemic wave- from 30 October to 15 December- and the third one – from 3 April to 2 May 2021. Furthermore, a curfew was in place between the second and third lockdowns because of a constant high circulation of SARS-CoV-2 in France. The dramatic decrease in medical visits during the first epidemic wave might have delayed and decreased the screening of arterial hypertension and other cardiovascular risk factors as well as hampered the initiation of related medication treatments as found in other studies (4, 5).

The main objective of this study was to analyze time-trends in the initiation of medication treatments for cardiovascular risk factors and diseases, such as antihypertensive and lipid-lowering treatments, smoking cessation medications and oral anticoagulants in patients with atrial fibrillation among the overall French population in 2020-2021 compared to 2017–2019. This first objective included the analysis of age and sex specific trends. A secondary objective was to analyze healthcare use during the same periods (family physician (general practitioner) and cardiologist visits, biological and clinical screening of cardiovascular risk factors).

## Materials and Methods

### Data Sources

Data from the French National Insurance Databases (“Système National des Données de Santé”[SNDS]) were used. This database records reimbursements of health care expenditure for all persons with French health insurance coverage, which corresponds approximately to the overall French population, i.e., approximatively 66 millions of inhabitants (6). All persons born in France, French or foreign, and legal immigrants are covered by a universal health insurance. More specifically, this database contains individual details of all reimbursed medication treatments delivered outside of hospitals. All cardiovascular medications are reimbursed in France. Individual socio-demographic data are also available such as date of birth, sex, or whether the person is covered by the Complementary Universal Health Insurance (CMUc), which provides free access to healthcare to people with a low annual income (6). As all care might not be reimbursed at 100%, people living in France have to subscribe to a complementary insurance. For people with low income, an equivalent of these complementary insurance – the CMUc – is granted for free.

### Population Selection and Cardiovascular Treatments

For each treatment of interest described below and for each year from 2017 to 2021, we selected all individuals in France (both metropolitan France and overseas territories) who had a first reimbursement for each medication treatment but no reimbursement in the past 12 months. As restriction measures were implemented in both metropolitan and overseas regions, the latter were included in the analysis and represented 3.2% of the study population. We used data until 23 May 2021 (week 20). Medication treatments for hypertension, dyslipidemia, smoking cessation and atrial fibrillation were considered, including antihypertensive, lipid-lowering agents (statins or others), smoking cessation products (nicotine replacement therapy (NRT) or varenicline), and oral anticoagulants when used for the prevention of thrombo-embolism in patients with atrial fibrillation (vitamin-K antagonist or direct oral anticoagulants). For antihypertensive medications, we first looked at the initiation of an antihypertensive medication whatever the class, and then according to the main classes of antihypertensive medications namely diuretics, betablockers, calcium channel blockers, angiotensin-converting enzyme (ACE) inhibitors and angiotensin II receptor blockers (ARBs). All these treatments were identified using “Anatomical Therapeutic Chemical Classification” (ATC) C02 except for C02CA02 (indoramin mainly used for migraine treatment), C03 (diuretics), C07 (betablocker), C08 (calcium channel blocker [CCB]), C09 (renin-angiotensin antagonist) for antihypertensives, N07BA (NRT, varenicline) for smoking cessation medications, C10 for lipid-lowering agents, B01AA (vitamin-K antagonist), B01AE07 (dabigatran), B01AF01 (rivaroxaban), and B01AF02 (Apixaban) for oral anticoagulant use in atrial fibrillation (edoxaban not yet commercialized in France). The use of oral anticoagulants in atrial fibrillation indication was determined using an algorithm described previously (7, 8). Briefly, the algorithm attributed the AF indication of oral anticoagulants regarding specific medical procedures, hospital diagnoses, delivery of other drugs and the presence of a competing cause (orthopedic surgery, venous thromboembolism, valvulopathy, etc…).

Some treatments, particularly antihypertensive medications, might not be prescribed for hypertension but in secondary prevention after an ischemic event for instance. Therefore we conducted complementary analyses by dissociating initiation of these medications in people with a history of cardiovascular disease from people without such history.

### Covariates

The following socio-demographics data were collected: age, sex, CMUc for people under 60 years, and whether the person lived in a nursing home. For those who initiated antihypertensive medication, lipid-lowering medication or smoking cessation medications, a history of cardiovascular disease was searched in the past 5 years according to the hospital diagnosis of cardiovascular disease or long-term disease status (LTD) using International Classification of Disease-10th revision (ICD-10) codes.

### Healthcare Consumptions Related to the Initiation of Cardiovascular Risk Treatments

In 2019 and 2020, medical visits with family physicians or cardiologists were recorded, with a limit of one visit per week per person. The number of teleconsultations with a family physician or a cardiologist was also distinguished. In France, teleconsultation has been authorized since June 2018. However, its use remained very low until the COVID-19 pandemic. Then, a 100% reimbursement of teleconsultations was acted in March 2020, prolonged until 2022. Furthermore, a phone-only teleconsultation was eligible to full reimbursement from April to May 2020.

For the years 2017–2021, reimbursements for blood sample analysis for lipid profile were searched using codes from the Nomenclature of Procedures in Laboratory Medicine and the use of Holter electrocardiogram (ECG) using codes from the French Medical Classification for Clinical Procedures. Two different measures were computed: the number of patients with at least one procedure in the year and the overall number of these procedures per year.

### Statistical Analysis

Using the national census population data provided by the National Institute of Statistics and Economic Studies, we calculated the rates of persons initiating the above-mentioned treatments of interest in France from 2017 to 2021. These rates were given by year, sex and age as national statistics gave us population census according to sex and age. Then, we estimated the annual incidence rate ratio (IRR) and weekly IRR between 2020–2021 and the previous time-period of 2017–2019 using Poisson regression adjusted for age and time trends from 2017 to 2019. In these regressions models, the census population was used as offset variable, and all were checked for over-dispersion. Models were stratified according to sex and age groups. We conducted a sensitivity analysis using an interrupted time series analysis to evaluate level changes introduced by the COVID-19 pandemic regarding the initiation of medication of interest. Results were detailed in Supplementary Material.

Statistical analyses were conducted using SAS Entreprise Guide 9.4.6.0.

### Ethics Approval

In line with French governmental regulations and the National Ethics Committee, no patient consent was required. The databases used in the study contained pseudonymized patient information. Furthermore, full access to the SNDS is granted to the National Agency for Public Health (Santé Publique France) by decree (regulatory decision DE-2011-078).

## Results

### Time Trends in the Initiation of Medical Treatment

In 2020, 1,518,686 persons initiated antihypertensive medication, 8,73,747 lipid-lowering medications, 2,27,409 oral anticoagulants in atrial fibrillation indication, and 6,96,351 smoking cessation medications (Table 1 and Supplementary Table 1). These numbers and the corresponding age-standardized rates were lower in 2020 than in 2019 regardless of the treatment (Table 1 and Supplementary Table 1). This observation was made for both men and women.

**TABLE 1:** Numbers and characteristics of people who initiated a therapy of interest between 2017 and 2021, and corresponding age-standardized rates in France.

	Antihypertensive medication					Lipid-lowering treatment					Oral anticoagulants for atrial fibrillation					Smoking cessation medications				
	2017	2018	2019	2020	2021*^a^*	2017	2018	2019	2020	2021^a^	2017	2018	2019	2020	2021^a^	2017	2018	2019	2020	2021^a^
**Number of persons initiating treatment**																				
	1,491,569	1,513,627	1,642,450	1,518,686	NA	817,259	800,615	896,118	873,747	NA	224,756	229,956	243,213	227,409	NA	327,872	675,257	865,697	696,351	NA
**Age-standardized*^b^* rates of persons initiating treatment**																				
All sexes	2.2%	2.3%	2.4%	2.2%	NA	1.2%	1.2%	1.3%	1.3%	NA	313.2*^c^*	310.6*^c^*	318.8*^c^*	309.1*^c^*	NA	0.5%	1.0%	1.3%	1.0%	NA
Men	2.1%	2.1%	2.2%	2.1%	NA	1.4%	1.3%	1.4%	1.3%	NA	408.8*^c^*	399.5*^c^*	392.6*^c^*	401.8*^c^*	NA	0.5%	1.0%	1.3%	1.1%	NA
Women	2.3%	2.4%	2.6%	2.4%	NA	1.1%	1.1%	1.3%	1.2%	NA	238.3*^c^*	240.5*^c^*	259.5*^c^*	237.1*^c^*	NA	0.5%	1.0%	1.3%	1.0%	NA
**Characteristics**																				
Mean age, years	56.7	57.3	58.4	57.9	58.1	62.6	63.2	64.1	63.9	63.9	75.3	75.2	75.2	75.0	75.0	47.5	47.7	46.9	46.9	47.5
Age groups,%																				
<45	23.2	22.1	20.3	21.1	20.7	8.9	8.2	7.3	7.5	7.2	2.1	2.2	2.2	2.4	2.5	40.6	39.6	42.4	43.1	41.8
45–64	42.7	42.4	41.2	41.4	41.5	45.7	44.5	42.3	42.6	42.8	15.6	15.6	15.4	15.8	15.9	49.1	49.2	47.0	46.1	46.2
65–74	18.6	19.2	20.9	20.8	20.9	25.9	27.0	28.8	29.1	29.2	25.0	25.3	25.7	26.2	25.8	8.9	9.6	9.0	9.2	10.1
75–84	10.1	10.6	11.9	11.1	11.4	14.0	14.6	15.9	15.1	15.3	31.6	31.1	30.9	30.1	30.1	1.3	1.5	1.4	1.5	1.8
≥85	5.4	5.6	5.8	5.6	5.6	5.5	5.7	5.8	5.7	5.5	25.7	25.9	25.7	25.4	25.7	0.1	0.1	0.2	0.2	0.2
Women,%	55.5	55.9	58.2	56.3	55.1	48.7	48.7	52.0	51.0	50.5	45.2	45.5	47.3	46.0	45.6	50.4	50.0	49.7	49.5	49.2
History of cardiovascular diseases,%	24.1	24.7	24.7	25.1	23.5	42.0	43.8	42.0	42.3	39.2	81.7	79.6	76.1	73.7	69.3	19.7	19.9	19.2	19.9	20.4
CMUc,%	11.3	11.1	11.0	11.9	12.6	11.2	11.0	10.7	11.7	12.4	7.2	6.8	7.1	7.9	8.7	7.6	11.6	13.3	14.8	16.5
Nursing home,%	12.7	12.4	11.3	5.7	4.9	13.7	13.4	12.2	6.2	5.5	17.5	17.1	16.3	9.8	8.8	6.0	8.8	10.1	4.5	3.7

*^a^Up to week 20 (23 May 2021); ^b^rates standardized based on the age structure of the 2017 French census population; ^c^rates per 100,000 inhabitants.*

*Italics: month of May incomplete for the year 2021. NA: not applicable; CMUc: Complementary Universal Medical Coverage.*

**FIGURE 1:**
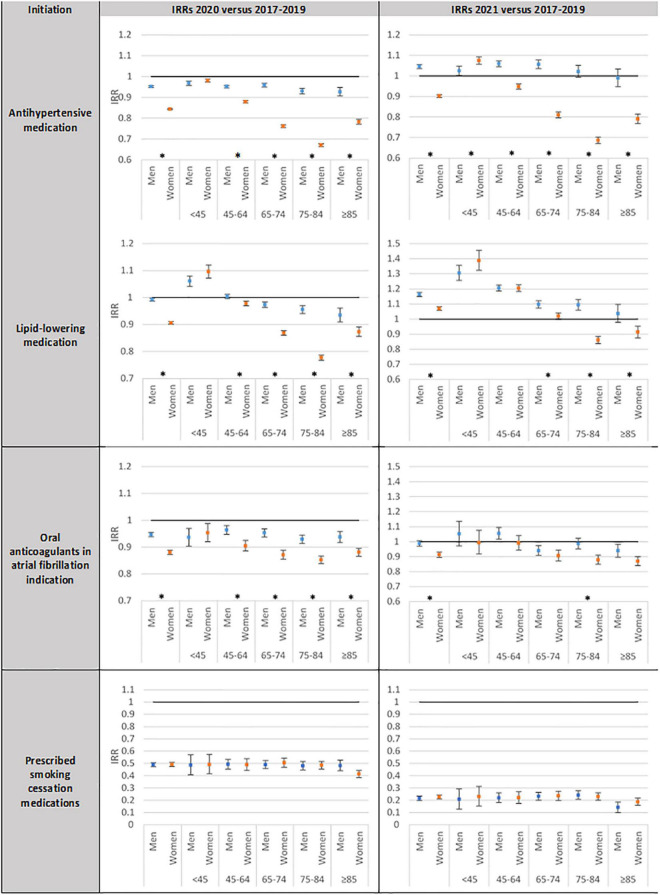
Incidence rate ratio (IRR)*^a^* and 95% confidence intervals (95% CI) between the rates of treatment initiation, respectively in 2020 and 2021 compared to the rates of initiation in 2017–2019 according to lockdown/curfew time-periods. Gray: lockdown period; light gray: curfew period. *^a^*adjusted for age and 2017–2019 time trends; W: weeks.

After accounting for age and 2017–2019 time trends, IRRs (Figure 1 and Supplementary Table 2) showed an overall decrease in the initiation of antihypertensive medications (−11.1%[CI95%, −11.4%;−10.8%]), lipid-lowering medications (−5.2%[CI95%, −5.5%;−4.8%]), oral anticoagulants for atrial fibrillation (−8.6%[CI95%, −9.1%;−8.0%]), and smoking cessation medications (−50.9%[CI95%, −51.1%;−50.7%]) in 2020 compared to 2017–2019. Regarding antihypertensive medications, a greater decrease was recorded for diuretics (−19.6%[CI95%, −20.1%; −19.1%]) and angiotensin-receptor blockers (ARBs) (−18.7[CI95%, −19.2%; −18.1%]) (data not shown).

During the first lockdown of 2020, the initiation of the treatments of interest fell sharply down (−30% for antihypertensive therapy, −38% for lipid-lowering medications, −70% for oral anticoagulants in atrial fibrillation indication, and −69% for smoking cessation medications) (Figure 1 and Supplementary Table 2). After the first lockdown, despite less marked decreases in the initiation of these treatments, the rates did not return to normal for the initiation of antihypertensive medications (−12%, respectively in weeks 20–43 and −3% in weeks 44–51 of 2020 compared to the same weeks in 2017–2019), oral anticoagulants in atrial fibrillation indication (−7% in weeks 20–43 and −3% in weeks 44–51) and smoking cessation medications (−50% for weeks 20–43 and 44–51). A significant increase in the initiation of lipid-lowering medications was nevertheless found for the second lockdown of 2020 (+ 8% compared to same weeks in 2017–2019) (Figure 1 and Supplementary Table 2).

In early 2021, different time-trends were observed with a lower decrease in the initiation of antihypertensive therapy in 2021 (−3.8%[CI95%, −4.4%;−3.2%]) than in 2020 (−11.1%[CI95%, −11.4%;−10.8%]) compared to 2017–2019 as well as for oral anticoagulant for atrial fibrillation (−4.9%[CI95%, −6.1%; −3.6%] in 2021 vs. −8.6%[CI95%, −9.1%;-−8.0%] in 2020) (Figure 1 and Supplementary Table 2). An increase in the initiation of lipid-lowering medications (+ 11.6%[CI95%, 10.7%;12.5%]) was found in 2021 compared to 2017–2019 whereas initiation of smoking cessation medications continued to decrease in 2021, increasing the gap with previous time period in 2017–2019 (−77.8%[CI95%, −78.1%;−77.6%]) (Figure 1 and Supplementary Table 2).

### Differences According to Sex, Age, and History of Cardiovascular Diseases

**FIGURE 2:**
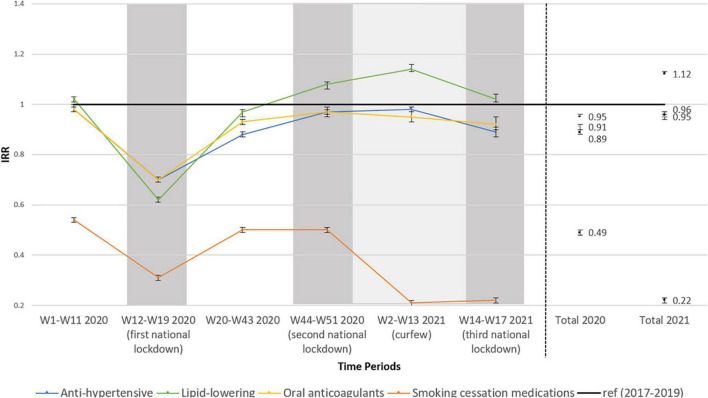
Incidence rate ratio (IRR)*^a^* and 95% confidence intervals (95% CI) between rates of initiation of treatments, respectively in 2020 and 2021 compared to the rates of initiation in 2017–2019 according to sex by age group. *^a^*adjusted for age and 2017–2019 time trends; IRRs: Incidence rate ratios; Blue: men; Orange: women.

When looking at the time trends in the initiation of the treatments of interest according to sex, age, and history of cardiovascular disease, substantial differences were observed (Figure 2 and Supplementary Figures 1–3).

First, according to sex, greater decreases were registered in women compared to men regarding the initiation of antihypertensive medication (−15.6%[CI95%, −15.9%; −15.3%] vs. −4.8%[CI95%, −5.2%; −4.4%], *p* < 0.0001), lipid-lowering medications (−9.4%[CI95%, −9.9%; −8.9%] vs. −0.8%[CI95%, −1.3%; −2.4%], *p* < 0.0001), and oral anticoagulants for atrial fibrillation (−11.9%[CI95%, −12.7%;−11.2%] vs. −5.3%[CI95%, −6.1%; −4.5%], *p* < 0.0001) between 2020 and 2017–2019 (Figure 2). These differences persisted in 2021 according to sex: the time trends between 2017–2019 and 2021 were + 4.5%[CI95%, 3.5%;5.4%] vs. −9.8%[CI95%, −10.6%; −9.1%] (*p* < 0.0001) for the initiation of antihypertensive medication, + 16.3%[CI95%, 14.9%; 17.6%] vs. + 7.0%[CI95%, 5.7%; 8.2%] (*p* < 0.0001) for lipid-lowering medications initiation, and −1.1%[CI95%, −2.9%;−0.7%] vs. −8.7%[CI95%, −10.4%; −7.0%] (*p* < 0.0001) for oral anticoagulants in atrial fibrillation indication for men and women, respectively. On the contrary, no difference between men and women was found for the initiation of smoking cessation medications.

Second, except for prescribed smoking cessation medications, the decrease in the initiation of the other treatments of interest between 2017–2019 and 2020 intensified with age, with the highest decrease recorded in people aged 75−84 years with −24.0%[CI95%, −24.7%; −23.4%] for antihypertensive medications, −15.4%[CI95%, −16.2%; −14.5%] for lipid-lowering medications, and −11.1%[CI95%, −12.1%; −10.0%] for oral anticoagulants in atrial fibrillation indication (Supplementary Figure 1). The differences observed between men and women also varied with age with higher differences in the elderly (Figure 2).

Third, the sex and age differences highlighted above were accentuated when looking at the initiation of treatments in people without a history of cardiovascular disease (−1.6%[CI95%, −2.1%; −1.1%] and −15.6%[CI95%, −15.9%; −15.2%] in men and women, respectively (*p* < 0.0001), for antihypertensive medication, and + 4.0%[CI95%, 3.2%; 4.9%] and −12.8%[CI95%, −13.4%; −12.2%] for lipid-lowering treatment (*p* < 0.0001)) (Supplementary Figure 2 and Supplementary Table 3).

### Time Trends of Other Healthcare-Related Uses

#### In-Patients Visits vs. Teleconsultations

Family physician and cardiologist visits declined by 8.4 and 7.4%, respectively, between 2019 and 2020 (Table 2). The largest decreases were found in people under 65 years for family physicians (−9.6%) and those aged over 85 years for cardiologists (−8.7%). Except in the under 65 age group, a greater decrease in family physician visits was found in women compared to men (respectively −5.1 and −3.9% in 65–74 age group, −7.5 and −5.8% in the 75–84 age group, and −5.8 and −4.1% in ≥85 age group). Furthermore, teleconsultations increased more in women aged 65–74 years compared to men of the same age group, reaching 4.2% of family physician visits in women aged 65–74 years between 2019 and 2020 vs. 4.0% in men of the same age, and 4.4 and 4.1% of family physician visits in women and men aged 75–84 years old, respectively (Table 2). By contrast, in these age groups, the number of in-patient family physician visits decreased more in women (−9.1 and −11.6%) than in men (−7.8 and −9.7%). Regarding cardiologist visits, the decrease between 2019 and 2020 was only greater in women compared to men over 75 years.

**TABLE 2:** Numbers and 2019–2020 time trends (%) of family physician and cardiology visits according to sex, age, and type of visit (in-person visit or teleconsultations).

		All			Men			Women		
		2019	2020	Trends	2019	2020	Trends	2019	2020	Trends
All	Family physician visits	256902333	235339827	−*8.4%*	106959033	98152850	−*8.2%*	149943300	137186977	−*8.5%*
	Family physician teleconsultations	88054	13133132		31872	5036430		56182	8096702	
	In-person family physician visits	256814279	222206695	−*13.5%*	106927161	93116420	−*12.9%*	149887118	129090275	−*13.9%*
	Cardiologist visits	11665744	10803323	−*7.4%*	6246327	5784830	−*7.4%*	5419417	5018493	−*7.4%*
	Cardiologist teleconsultations	619	68984		321	37168		298	31816	
	In-person cardiologist visits	11665125	10734339	−*8.0%*	6246006	5747662	−*8.0%*	5419119	4986677	−*8.0%*

<65	Family physician visits	179511055	162188615	−*9.6%*	75636371	68265123	−*9.7%*	103874684	93923492	−*9.6%*
	Family physician teleconsultations	77451	9977869		27539	3790710		49912	6187159	
	In-person family physician visits	179433604	152210746	−*15.2%*	75608832	64474413	−*14.7%*	103824772	87736333	−*15.5%*
	Cardiologist visits	4811191	4432193	−*7.9%*	2619744	2398400	−*8.4%*	2191447	2033793	−*7.2%*
	Cardiologist teleconsultations	321	27377		188	14803		133	12574	
	In-person cardiologist visits	4810870	4404816	−*8.4%*	2619556	2383597	−*9.0%*	2191314	2021219	−*7.8%*

65–74	Family physician visits	36232730	34564327	−*4.6%*	16075597	15443282	−*3.9%*	20157133	19121045	−*5.1%*
	Family physician teleconsultations	4735	1411667		2307	615987		2428	795680	
	In-person family physician visits	36227995	33152660	−*8.5%*	16073290	14827295	−*7.8%*	20154705	18325365	−*9.1%*
	Cardiologist visits	3270387	3080580	−*5.8%*	1862606	1752099	−*5.9%*	1407781	1328481	−*5.6%*
	Cardiologist teleconsultations	94	19018		63	10988		31	8030	
	In-person cardiologist visits	3270293	3061562	−*6.4%*	1862543	1741111	−*6.5%*	1407750	1320451	−*6.2%*

75–84	Family physician visits	25525998	23781618	−*6.8%*	10411888	9807779	−*5.8%*	15114110	13973839	−*7.5%*
	Family physician teleconsultations	3047	1020489		1213	406943		1834	613546	
	In-person family physician visits	25522951	22761129	−*10.8%*	10410675	9400836	−*9.7%*	15112276	13360293	−*11.6%*
	Cardiologist visits	2508825	2309188	−*8.0%*	1302071	1206026	−*7.4%*	1206754	1103162	−*8.6%*
	Cardiologist teleconsultations	79	15313		37	8157		42	7156	
	In-person cardiologist visits	2508746	2293875	−*8.6%*	1302034	1197869	−*8.0%*	1206712	1096006	−*9.2%*

≥85	Family physician visits	15632550	14805267	−*5.3%*	4835177	4636666	−*4.1%*	10797373	10168601	−*5.8%*
	Family physician teleconsultations	2821	723107		813	222790		2008	500317	
	In-person family physician visits	15629729	14082160	−*9.9%*	4834364	4413876	−*8.7%*	10795365	9668284	−*10.4%*
	Cardiologist visits	1075341	981362	−*8.7%*	461906	428305	−*7.3%*	613435	553057	−*9.8%*
	Cardiologist teleconsultations	125	7276		33	3220		92	4056	
	In-person cardiologist visits	1075216	974086	−*9.4%*	461873	425085	−*8.0%*	613343	549001	−*10.5%*

#### Lipid Blood Tests

The rates of persons who had at least one reimbursement for lipid blood profiling decreased by −5.3%[CI95%, −5.4%; −5.3%] between 2017–2019 and 2021, similarly in men and women (Table 3 and Supplementary Table 4). A larger decrease was observed among people aged under 45 years (−9.3%[CI95%, −9.5; −9.2%]) compared to the eldest age group over 85 years (−2.7%[CI95%, −3.0%; −2.4%]). In 2021, the rates were similar as compared to 2017–2019 in both men and women for all age groups.

**TABLE 3:** Incidence rate ratio (IRR)* and 95% confidence intervals (95% CI) for the rates of people with at least one lipid blood test and one Holter ECG in 2020 and 2021 compared to the rates in 2017–2019.

		Lipid blood test		Holter ECG	
		2020	2021 (W1-W20)	2020	2021 (W1-W20)
Total		0.94[0.94–0.94]	1.01[1.01–1.01]	0.97[0.97–0.98]	1.07[1.06–1.08]
Men		0.94[0.94–0.94]	0.99[0.99–0.99]	0.98[0.97–0.98]	1.06[1.05–1.07]
Women		0.95[0.94–0.95]	1.03[1.02–1.03]	0.97[0.97–0.98]	1.08[1.06–1.09]
<45		0.90[0.90–0.90]	1.00[1.00–1.00]	0.92[0.91–0.93]	1.03[1.01–1.05]
45–64		0.94[0.93–0.94]	1.03[1.03–1.03]	0.95[0.94–0.95]	1.07[1.05–1.08]
65–74		0.97[0.97–0.97]	0.99[0.99–1.00]	0.96[0.95–0.97]	1.04[1.02–1.05]
75–84		0.97[0.97–0.97]	1.01[1.01–1.02]	0.99[0.98–1.00]	1.08[1.07–1.10]
≥85		0.98[0.98–0.98]	0.99[0.98–1.00]	1.13[1.11–1.14]	1.17[1.14–1.19]
< 45	Men	0.88[0.87–0.88]	0.96[0.96–0.97]	0.93[0.92–0.95]	1.02[0.99–1.05]
	Women	0.91[0.91–0.92]	1.02[1.01–1.02]	0.91[0.90–0.93]	1.03[1.00–1.06]
45–64	Men	0.93[0.93–0.93]	1.01[1.01–1.01]	0.97[0.96–0.98]	1.09[1.07–1.11]
	Women	0.94[0.94–0.94]	1.05[1.04–1.05]	0.93[0.92–0.94]	1.05[1.03–1.07]
65–74	Men	0.97[0.97–0.97]	0.97[0.97–0.98]	0.97[0.96–0.98]	1.03[1.01–1.05]
	Women	0.97[0.97–0.98]	1.01[1.01–1.02]	0.96[0.95–0.97]	1.04[1.02–1.06]
75–84	Men	0.97[0.96–0.97]	1.00[0.99–1.00]	0.98[0.97–0.99]	1.05[1.03–1.08]
	Women	0.97[0.97–0.97]	1.03[1.02–1.03]	1.00[0.98–1.01]	1.11[1.09–1.14]
≥85	Men	0.97[0.97–0.98]	0.98[0.97–0.99]	1.08[1.06–1.10]	1.12[1.08–1.16]
	Women	0.98[0.98–0.99]	1.00[0.99–1.00]	1.16[1.14–1.18]	1.20[1.16–1.24]

* adjusted for age and 2017–2019 time trends.

#### Holter Electrocardiogram

The use of Holter ECG globally decreased by −3.0%[CI95%, −3.4%; −2.7%] in 2020 compared to 2017–2019, with a greater decrease among people aged under 45 years (−7.9%[CI95%, −8.7; −7.0%]) and an increase observed in those aged over 85 years (+ 10.4%[CI95%, 9.2; 11.6%]) (Table 3 and Supplementary Table 4). The use of Holter ECG increased in 2021 compared to 2017–2019 (+ 6.8%[CI95%, 6.0%; 7.6%]).

## Discussion

A marked decrease in the initiation of cardiovascular risk main treatments was observed during the first year of the COVID-19 pandemic. Women and the oldest age groups seemed to be more impacted, particularly regarding the initiation of antihypertensive medication. Although a catch-up effect might be seen for lipid-lowering medications or in the youngest age groups in 2021, the initiation rates for other treatment of the cardiovascular risk remained generally lower in 2021 compared to time-period prior to the COVID-19 pandemic, especially in women. Concomitantly, a decrease in family physician and cardiologist visits along with a surge in teleconsultations was found with variations according to sex and age.

The implications of the COVID-19 pandemic regarding the incidence and management of cardiovascular diseases and their risk factors was predictable (5). Overall, this could be caused by a decrease in healthcare visits during the pandemic, leading to a decrease in the screening of hypertension, hypercholesterolemia and atrial fibrillation, and a less consideration of smoking. The initiation of antihypertensive medication and oral anticoagulants in atrial fibrillation was indeed the most impacted according to our results. Alexander et al. found the large decreases in blood pressure (−50.1%) and cholesterol level assessments (−36.9%) in 2020 compared to 2018–2019 in the United States, highlighting that these assessments were significantly less common during telemedicine than during in-person visits (9.6% vs. 69.7% for blood pressure; 13.5% vs. 21.6% for cholesterol) (4). Telehealth visits were associated with fewer new medication prescriptions in another American study examining how the pandemic impacted outpatient cardiology care (9). Although people could access telehealth consults instead of in-person visits with a more equitable access to healthcare, many patients did not visit a medical doctor at all. The increase in telehealth consultation during the COVID-19 pandemic did not compensate the decrease of in-person visits.

At the beginning of the pandemic, it was hypothesized that angiotensin-converting-enzyme (ACE) inhibitors and ARB medications was associated with a higher risk and severity of infection by SARS-CoV-2. Several studies thereafter showed the absence of an association or the lower risk or severity of SARS-CoV-2 infection in patients treated with these medications (10–13), including in France (14). Our results suggested no clear impact of this controversy on the initiation of ARB and ACE inhibitors medications that could be related to the quick denials of cardiology societies.

The lower rates for the initiation of oral anticoagulants for atrial fibrillation during the pandemic was observed elsewhere (15, 16) and was consistent with the decrease in both family physician and cardiology visits as well as the use of Holter ECG. The increased use of teleconsultations might have been particularly harmful for the opportunistic screening of asymptomatic hypertension or atrial fibrillation. Although teleconsultation was well accepted during the pandemic, it does not allow a proper clinical examination, with a risk of underdiagnosis and low rates of treatment initiation as reported elsewhere (17, 18). Regarding hypertension, no specific recommendation on the use of telehealth services for the screening was mentioned in the latest recommendations from the societies of Hypertension. Only an international consensus from the World Heart Federation was published (19). This position paper remained very general and reported telehealth experiences more in the management of already diagnosed hypertension than in the screening. Regarding AF, strategies of heart rate and rhythm monitoring through teleconsultation were spread in order to allow the screening of atrial fibrillation despite the COVID-19 pandemic, namely TeleCheck-AF (20, 21). An increase by 20% in the number of ECG with remote transmission was observed in 2020 compared to 2019 (data not shown). However, TeleCheck-AF approach was mainly developed to remotely detect AF episodes in patients who have been diagnosed for AF by ECG before. A drop in the initiation of the use of prescribed smoking cessation medications was observed in our study with no rebound in 2021. The drop took into account the time-trend before the pandemic which showed almost a doubling in the initiation of prescribed smoking cessation medications between 2017 and 2019. Between 2017 and 2018, the doubling could be related to the national prevention campaign “Tobacco-Free Month” launched in October 2016 (22) with particularly substantial increase during the month of the campaign (data not shown). Between 2018 and 2019, the time-trend could also be related to a change in the reimbursement of these smoking cessation medications which have become fully reimbursed since, when prescribed by a physician. On the contrary the French Monitoring Centre for Drugs and Drug Addiction (OFDT) showed an increase by 4% of smoking cessation medications (delivered OTC or prescribed) in 2020 compared to 2019 (23). Therefore, our study showed a substantial impact of the pandemic on prescribed smoking cessation medication that could be related to the decrease in physician visits. This decrease might have been compensated by the increase in OTC smoking cessation medications. The effectiveness of NRT (the most sold of the smoking cessation medication) being higher when prescribed by a physician, the increase in OTC medication sales may be associated with a lower rate of effective smoking cessation in France in 2020. A British study carried out during the 2006–2018 period showed that the use of OTC NRTs was not related with self-reported tobacco abstinence, contrary to prescribed ones (24). Thus, the increase in OTC medication sales may be associated with a lower rate of effective smoking cessation in France in 2020. Several studies showed an increase in tobacco use among current smokers (25–27), including in France (28), and a decrease in the motivation to quit (29). A US study also showed a dramatic decrease in the use of quit lines (30). Nevertheless, these changes mainly affected adults under 50 years of age in France (28). The polemic appeared in May 2020 around the potential protective effect of nicotine against SARS-CoV-2 infection and severity did not seem to have led to an increase in reimbursed smoking cessation medications. The government had limited the delivery of smoking cessation medications by fear of a rush to drugstore following this polemic. Finally, OFDT showed a massive increase in oral form of smoking cessation medications instead of nicotine patch.

The greater decrease in the initiation of treatments observed in older women aged 65–84 compared to men of the same age group between 2017–2019 and 2020 could relate to the larger decrease in family physician visits alongside the higher use of teleconsultations in women compared to men of this age group. This observation highlighted the different healthcare attendance and use of telemedicine during the pandemic according to age and sex. A study in the United States showed higher use of telemedicine among women during the pandemic compared to men (31). A decrease in gynecology visits has been observed in France in 2020 compared to 2019 and could be related to the higher decrease in women even in older age groups (data not shown).

Finally, we cannot exclude that the real incidence of cardiovascular risk factors decreased since the beginning of the pandemic due to change in people’s behavior or diet and a reduction of professional and other stress. A study showed a decrease in blood pressure during the first lockdown in France (32). However, it seems less likely that these changes would have so quickly impacted blood pressure, lipid profile, or atrial fibrillation incidence. On the contrary, several studies showed an increase in sedentary behaviors, a decrease in physical activity and an increase in depression rates during the pandemic (33–36), which could lead to an increase in the incidence of cardiovascular risk factors (37), with the same limitations between the exposure to such behaviors and the incidence of arterial hypertension, hypercholesterolemia, or atrial fibrillation. Furthermore, studies on the impact of the COVID-19 pandemic on behavioral determinants of health revealed mitigated results (38–44) and were limited to the first epidemic wave, although the persistence of this impact has not been explored. We cannot also exclude that a decrease in the incidence of cardiovascular risk factors could be related to the death of patients at high cardiovascular risk because of SARS-CoV-2 infection. The decrease in overall cardiology procedures in 2020 vs. 2019 (data not shown) might indeed be due to a decrease in cardiovascular patients independently of the decrease in the number of cardiology visits.

A “catch-up” effect could be hypothesized for the initiation of lipid-lowering medications in the first months of 2021 although a lesser increase in lipid blood test rates was found. This could be also explained by the increase in visits to a cardiologist and an endocrinologist that has been found during the second lockdown whereas rates of visits to a family physician continue to decrease at that time (data not shown). The increase in rates of initiation of lipid-lowering medications in 2021 could also be related to constant increase in the use of these medications due to changes in the guidelines for circulating cholesterol concentrations achievement toward always reduced cholesterol levels. The latest guidelines dated from 2019 (23). Furthermore, no rush to visit a family physician or a cardiologist was observed before the first lockdown in France on the contrary to a marked stock of medical treatments. No “catch-up” effect was found for the other medications. This might be explained by the continuous decrease in family physician visits. It also suggested that the “catch-up” effect for lipid-lowering medication might be related to the latest guidelines, a specific change for these medications only.

### Clinical and Public Health Implications

These findings have important implications in the field of cardiovascular disease prevention. There could be a larger pool of patients at risk of cardiovascular diseases today compared to the pre-pandemic time period. The population was differentially impacted according to sex and age, which imply the need for sex-specific prevention. Women over 65 years old seemed to pay a high price during the COVID-19 pandemic, although this could be related to a decline in the screening of cardiovascular diseases in women. The Lancet commission on women and cardiovascular diseases alerts about “the stagnation on the overall reduction of cardiovascular disease burden for women in the past decade,” as “cardiovascular disease in women remains understudied, under-recognized, underdiagnosed, and undertreated” (45). Greater attention should also be paid to people who used teleconsultations during the pandemic or had fewer medical visits.

### Limitations

This study analyzed main treatments for cardiovascular risk and diseases, and therefore only the treated patients with arterial hypertension, hypercholesterolemia, or atrial fibrillation. The initiation of some antihypertensive medications could also be related to a cardiovascular event and not hypertension itself. To deal with this confusion, we studied the initiation of medications, respectively in people with a history of cardiovascular diseases and in people without such history. Almost all reimbursements of these treatments were recorded with the exception of treatments delivered in nursing homes with an in-house pharmacy. Nevertheless, only 21% of nursing homes have an in-house pharmacy in France, and a very small part of our treatment of interest was initiated when the patient was already in these healthcare structures (46). Furthermore, approximately 600,000 persons live in medical institutions in France, equivalent to less than 1% of the population. Regarding smoking cessation medications, these treatments are not exhaustively registered in our database as an unknown proportion is sold without prescription. Our analysis did not take into account OTC medications for hypertension, hypercholesterolemia or atrial fibrillation which are very rare in France for these conditions. OTC smoking cessation medications are on the contrary usually used in France. Therefore our study only reflected the impact of the COVID-19 pandemic on prescribed smoking cessation medications and therefore physician visits. Finally, antidiabetic medications were not included in our study despite diabetes put patients at high cardiovascular risk, because of a current study lead specifically for this treatment.

## Conclusion

The pandemic had a major impact on the initiation of cardiovascular medication treatments and therefore the incidence and/or screening of cardiovascular risk factors, particularly in women, which could have consequences on the incidence of cardiovascular diseases in the near future. Lower rates of treatment initiations were found in 2021 compared to the period prior to the pandemic for antihypertensive medications and women aged over 65 years. The effect of the exponential use of teleconsultations on the screening and management of cardiovascular risk factors should be the focus of further research as well as the decrease in the overall number of physician visits which was not compensated after the first national lockdown.

## Data Availability Statement

The original contributions presented in the study are included in the article/Supplementary Material, further inquiries can be directed to the corresponding author.

## Ethics Statement

Ethical review and approval was not required for the study on human participants in accordance with the local legislation and institutional requirements. Written informed consent from the participants’ legal guardian/next of kin was not required to participate in this study in accordance with the national legislation and the institutional requirements.

## Author Contributions

AG directed the research, wrote the manuscript, and performed the statistical analyses. CGr, TL, VN-T, and RG contributed to statistical analyses, discussion and reviewed the manuscript. PT, CGu, JB, and VO directed the research, contributed to discussion and reviewed the manuscript. All authors contributed to the article and approved the submitted version.

## Conflict of Interest

CGu reported, outside the submitted work, grants from Microport CRM, consulting fees from Boston Scientific and Microport CRM, and honoraria from Medtronic. JB reports, outside the submitted work, personal fees from Abbott, Bayer, Bottu, Ferring, Steripharma, Kantar, Teriak, personal fees and non-financial support from Pfizer, Quantum Genomics, personal fees from Sanofi and Servier. The remaining authors declare that the research was conducted in the absence of any commercial or financial relationships that could be construed as a potential conflict of interest.

## Publisher’s Note

All claims expressed in this article are solely those of the authors and do not necessarily represent those of their affiliated organizations, or those of the publisher, the editors and the reviewers. Any product that may be evaluated in this article, or claim that may be made by its manufacturer, is not guaranteed or endorsed by the publisher.
