# Supplementary material for: Nationwide Initiation of Cardiovascular Risk Treatments During the COVID-19 Pandemic in France: Women on a Slippery Slope?
Source: Front Cardiovasc Med. 2022 Apr 25;9:856689. doi: 10.3389/fcvm.2022.856689 (PMC9081923; doi:10.3389/fcvm.2022.856689)
Supplement: Supplementary file 1 [file Data_Sheet_1.DOCX]

# Supplemental Data

e-Table 1: Numbers of people who initiated a medication treatment of interest according to years and months from 2017 to 2021 in France

|  | **Antihypertensive treatment** | | | | |  | **Lipid-lowering treatment** | | | | |  | **Oral anticoagulants for atrial fibrillation** | | | | |  | **Nicotine replacement therapy** | | | | |  |
| --- | --- | --- | --- | --- | --- | --- | --- | --- | --- | --- | --- | --- | --- | --- | --- | --- | --- | --- | --- | --- | --- | --- | --- | --- |
|  | **2017** | **2018** | **2019** | **2020** | **2021^a^** |  | **2017** | **2018** | **2019** | **2020** | **2021 ^a^** |  | **2017** | **2018** | **2019** | **2020** | **2021 ^a^** |  | **2017** | **2018** | **2019** | **2020** | **2021 ^a^** |  |
| **Numbers of persons who initiated treatment** | | | | | | | | | | | | | | | | | | | | | | | |  |
| - **January** | 138901 | 140060 | 150030 | 147358 | 145844 |  | 67839 | 69862 | 76011 | 77457 | 84048 |  | 20633 | 21049 | 21501 | 21728 | 19438 |  | 30571 | 38336 | 94416 | 86751 | 63543 |  |
| - **February** | 126433 | 128844 | 135288 | 137531 | 140245 |  | 67060 | 67088 | 71932 | 76800 | 85486 |  | 18992 | 19243 | 20310 | 20543 | 18651 |  | 24607 | 31025 | 73768 | 73258 | 57115 |  |
| - **March** | 148002 | 146676 | 147524 | 131726 | 165719 |  | 82292 | 76076 | 80077 | 68718 | 100888 |  | 21313 | 21772 | 21402 | 18569 | 21653 |  | 25585 | 38593 | 83310 | 64897 | 63993 |  |
| - **April** | 118879 | 126391 | 146056 | 99873 | 149118 |  | 65563 | 65622 | 78350 | 44852 | 90135 |  | 17778 | 19133 | 21202 | 14032 | 19979 |  | 19586 | 34934 | 70830 | 38062 | 55679 |  |
| - **May** | 128934 | 120846 | 142859 | 110310 | *134463* |  | 70647 | 61922 | 78195 | 61536 | *81770* |  | 19024 | 18652 | 20838 | 16909 | *18391* |  | 22275 | 37486 | 62759 | 39242 | *48511* |  |
| - **June** | 119832 | 124011 | 131889 | 130225 | NA |  | 69809 | 68359 | 75064 | 78724 | NA |  | 18495 | 19291 | 19397 | 20046 | NA |  | 23363 | 62218 | 57755 | 50063 | NA |  |
| - **July** | 105593 | 107430 | 132810 | 119281 | NA |  | 61782 | 59436 | 76225 | 74274 | NA |  | 16788 | 17533 | 20262 | 19367 | NA |  | 22462 | 50937 | 57995 | 49248 | NA |  |
| - **August** | 91596 | 88871 | 105394 | 93181 | NA |  | 51408 | 47254 | 57673 | 56660 | NA |  | 15278 | 15061 | 16794 | 15532 | NA |  | 21403 | 50953 | 49889 | 42842 | NA |  |
| - **September** | 119437 | 113436 | 127870 | 126143 | NA |  | 67666 | 61550 | 72132 | 78678 | NA |  | 17901 | 17370 | 18558 | 19354 | NA |  | 29467 | 72459 | 69425 | 57894 | NA |  |
| - **October** | 131811 | 142876 | 147480 | 141732 | NA |  | 73952 | 76792 | 80898 | 85289 | NA |  | 19561 | 21151 | 21884 | 21176 | NA |  | 33306 | 91664 | 82667 | 63715 | NA |  |
| - **November** | 134247 | 141491 | 140674 | 139496 | NA |  | 72657 | 74744 | 74803 | 83773 | NA |  | 19390 | 19979 | 20194 | 19315 | NA |  | 40979 | 94027 | 93658 | 72585 | NA |  |
| - **December** | 127904 | 132695 | 134576 | 141830 | NA |  | 66584 | 71910 | 74758 | 86986 | NA |  | 19604 | 19723 | 20872 | 20839 | NA |  | 34268 | 72625 | 69225 | 57794 | NA |  |

* Up to week 20 (23 May 2021); ^b^rates standardized based on the age structure of the 2017 French census population; ^c^rates per 100,000 inhabitants.

Italics: month of May incomplete for the year 2021.

NA: not applicable

e-Table 2: Incidence rate ratio (IRR)* and 95% confidence intervals (95% CI) between the rates of treatment initiation respectively in 2020 and 2021 compared to the rates of initiation in 2017-2019 **according to lockdown/curfew time-periods**

|  |  | **2020 vs 2017-2019** | | | | | | | | |  | **2021 vs 2017-2019** | | | | |  |
| --- | --- | --- | --- | --- | --- | --- | --- | --- | --- | --- | --- | --- | --- | --- | --- | --- | --- |
| **IRR[95% CI]** |  | **W1-W11** |  | **W12-W19 (first national lockdown)** |  | **W20-W43** |  | **W44-W51 (second national lockdown)** |  | **Total 2020** |  | **W2-W13 (curfew)** |  | **W14-W17(third national lockdown)** |  | **Total 2021** |  |
| **Anti-hypertensive** |  | 0.98[0.97-0.98] |  | 0.70[0.69-0.70] |  | 0.88[0.87-0.88] |  | 0.97[0.96-0.98] |  | 0.89[0.89-0.89] |  | 0.98[0.97-0.99] |  | 0.89[0.87-0.9] |  | 0.96[0.96-0.97] |  |
| **Lipid-lowering** |  | 1.02[1.01-1.03] |  | 0.62[0.62-0.63] |  | 0.97[0.96-0.98] |  | 1.08[1.07-1.09] |  | 0.95[0.94-0.95] |  | 1.14[1.12-1.15] |  | 1.02[1.00-1.04] |  | 1.12[1.11-1.13] |  |
| **Oral anticoagulants** |  | 0.98[0.97-1.00] |  | 0.70[0.69-0.71] |  | 0.93[0.92-0.94] |  | 0.97[0.96-0.99] |  | 0.91[0.91-0.92] |  | 0.95[0.94-0.97] |  | 0.92[0.90-0.95] |  | 0.95[0.94-0.96] |  |
| **Nicotine replacement therapy** |  | 0.54[0.53-0.54] |  | 0.31[0.31-0.32] |  | 0.50[0.49-0.50] |  | 0.50[0.50-0.51] |  | 0.49[0.49-0.49] |  | 0.21[0.21-0.21] |  | 0.22[0.22-0.23] |  | 0.22[0.22-0.22] |  |

*adjusted for age and 2017-2019 time trends; W: weeks.

e-Table 3: Incidence rate ratio (IRR)* between the treatment initiation in 2020 and 2021 respectively versus 2017-2019 **according to previous history of cardiovascular diseases.**

|  | |  | **History of cardiovascular diseases** | | |  | **No history of cardiovascular diseases** | | |  |
| --- | --- | --- | --- | --- | --- | --- | --- | --- | --- | --- |
|  |  |  | **2020** |  | **2021 (W1-W20)** |  | **2020** |  | **2021 (W1-W20)** |  |
| ***Initiation of antihypertensive medication*** | | | | | | | | | | |
| **Total** |  |  | 0.89[0.89-0.90] |  | 0.86[0.85-0.87] |  | 0.90[0.89-0.90] |  | 0.97[0.96-0.97] |  |
| - **Men** |  |  | 0.93[0.93-0.94] |  | 0.92[0.90-0.93] |  | 0.98[0.98-0.99] |  | 1.08[1.07-1.10] |  |
| - **Women** |  |  | 0.84[0.84-0.85] |  | 0.79[0.77-0.80] |  | 0.84[0.84-0.85] |  | 0.89[0.88-0.90] |  |
| - **<45** |  |  | 0.94[0.92-0.96] |  | 0.90[0.86-0.94] |  | 1.00[0.99-1.01] |  | 1.07[1.05-1.08] |  |
| - **45-64** |  |  | 0.91[0.90-0.92] |  | 0.88[0.86-0.90] |  | 0.93[0.92-0.93] |  | 1.00[0.99-1.01] |  |
| - **65-74** |  |  | 0.89[0.88-0.90] |  | 0.86[0.84-0.88] |  | 0.83[0.82-0.83] |  | 0.90[0.89-0.92] |  |
| - **75-84** |  |  | 0.84[0.83-0.86] |  | 0.82[0.79-0.84] |  | 0.70[0.69-0.71] |  | 0.74[0.72-0.75] |  |
| - **≥85** |  |  | 0.88[0.86-0.89] |  | 0.84[0.81-0.87] |  | 0.78[0.76-0.79] |  | 0.81[0.78-0.85] |  |
| **<45** | **Men** |  | 0.95[0.93-0.98] |  | 0.93[0.87-0.99] |  | 1.00[0.98-1.01] |  | 1.05[1.02-1.07] |  |
|  | **Women** |  | 0.92[0.89-0.95] |  | 0.86[0.80-0.92] |  | 1.00[0.99-1.01] |  | 1.07[1.06-1.09] |  |
| **45-64** | **Men** |  | 0.94[0.93-0.95] |  | 0.91[0.89-0.94] |  | 0.98[0.97-0.98] |  | 1.09[1.07-1.10] |  |
|  | **Women** |  | 0.87[0.85-0.88] |  | 0.81[0.78-0.84] |  | 0.89[0.88-0.89] |  | 0.94[0.92-0.95] |  |
| **65-74** | **Men** |  | 0.94[0.92-0.95] |  | 0.92[0.89-0.96] |  | 0.99[0.98-1.01] |  | 1.10[1.08-1.13] |  |
|  | **Women** |  | 0.83[0.82-0.85] |  | 0.77[0.74-0.80] |  | 0.74[0.73-0.74] |  | 0.78[0.77-0.80] |  |
| **75-84** | **Men** |  | 0.91[0.89-0.93] |  | 0.92[0.88-0.96] |  | 0.97[0.95-0.99] |  | 1.10[1.06-1.15] |  |
|  | **Women** |  | 0.78[0.77-0.80] |  | 0.73[0.70-0.76] |  | 0.61[0.60-0.61] |  | 0.60[0.59-0.62] |  |
| **≥85** | **Men** |  | 0.92[0.90-0.95] |  | 0.90[0.85-0.95] |  | 0.98[0.94-1.02] |  | 1.12[1.04-1.21] |  |
|  | **Women** |  | 0.85[0.83-0.87] |  | 0.81[0.77-0.84] |  | 0.72[0.71-0.74] |  | 0.73[0.70-0.76] |  |
| ***Initiation of lipid-lowering medication*** | | | | | | | | | | |
| **Total** |  |  | 0.94[0.93-0.94] |  | 0.97[0.96-0.98] |  | 0.94[0.94-0.95] |  | 1.18[1.17-1.19] |  |
| - **Men** |  |  | 0.94[0.93-0.95] |  | 0.97[0.96-0.99] |  | 1.04[1.03-1.05] |  | 1.32[1.30-1.35] |  |
| - **Women** |  |  | 0.93[0.92-0.94] |  | 0.96[0.94-0.98] |  | 0.87[0.87-0.88] |  | 1.08[1.06-1.09] |  |
| - **<45** |  |  | 1.01[0.97-1.04] |  | 1.08[1.00-1.16] |  | 1.09[1.08-1.11] |  | 1.37[1.33-1.42] |  |
| - **45-64** |  |  | 0.95[0.94-0.96] |  | 0.99[0.97-1.01] |  | 1.01[1.00-1.01] |  | 1.29[1.27-1.31] |  |
| - **65-74** |  |  | 0.93[0.92-0.94] |  | 0.93[0.91-0.95] |  | 0.88[0.87-0.89] |  | 1.09[1.07-1.11] |  |
| - **75-84** |  |  | 0.92[0.91-0.93] |  | 0.97[0.94-1.00] |  | 0.73[0.72-0.74] |  | 0.87[0.84-0.90] |  |
| - **≥85** |  |  | 0.94[0.92-0.95] |  | 0.97[0.93-1.01] |  | 0.77[0.74-0.80] |  | 0.91[0.85-0.98] |  |
| **<45** | **Men** |  | 1.02[0.98-1.06] |  | 1.07[0.98-1.17] |  | 1.07[1.05-1.10] |  | 1.35[1.29-1.41] |  |
|  | **Women** |  | 0.98[0.92-1.04] |  | 1.10[0.97-1.26] |  | 1.12[1.10-1.15] |  | 1.42[1.34-1.49] |  |
| **45-64** | **Men** |  | 0.95[0.94-0.96] |  | 0.99[0.96-1.02] |  | 1.04[1.03-1.05] |  | 1.33[1.30-1.36] |  |
|  | **Women** |  | 0.95[0.93-0.97] |  | 0.99[0.95-1.03] |  | 0.97[0.96-0.98] |  | 1.24[1.21-1.27] |  |
| **65-74** | **Men** |  | 0.93[0.92-0.94] |  | 0.93[0.90-0.96] |  | 1.03[1.01-1.04] |  | 1.28[1.23-1.33] |  |
|  | **Women** |  | 0.92[0.91-0.94] |  | 0.93[0.89-0.97] |  | 0.81[0.80-0.82] |  | 0.99[0.96-1.02] |  |
| **75-84** | **Men** |  | 0.93[0.92-0.95] |  | 0.99[0.95-1.03] |  | 1.01[0.98-1.04] |  | 1.33[1.25-1.42] |  |
|  | **Women** |  | 0.90[0.89-0.92] |  | 0.96[0.92-1.00] |  | 0.64[0.63-0.65] |  | 0.71[0.69-0.74] |  |
| **≥85** | **Men** |  | 0.93[0.90-0.96] |  | 0.99[0.93-1.06] |  | 1.02[0.95-1.09] |  | 1.37[1.18-1.59] |  |
|  | **Women** |  | 0.94[0.92-0.96] |  | 0.96[0.91-1.01] |  | 0.71[0.68-0.74] |  | 0.80[0.74-0.87] |  |
| ***Initiation of nicotine replacement therapy*** | | | | | | | | | | |
| **Total** |  |  | 0.49[0.49-0.50] |  | 0.20[0.20-0.21] |  | 0.47[0.46-0.47] |  | 0.18[0.18-0.18] |  |
| - **Men** |  |  | 0.50[0.49-0.51] |  | 0.21[0.21-0.22] |  | 0.46[0.46-0.47] |  | 0.17[0.17-0.18] |  |
| - **Women** |  |  | 0.48[0.48-0.49] |  | 0.19[0.18-0.20] |  | 0.47[0.47-0.47] |  | 0.19[0.18-0.19] |  |
| - **<45** |  |  | 0.49[0.47-0.50] |  | 0.19[0.18-0.20] |  | 0.47[0.47-0.48] |  | 0.19[0.18-0.19] |  |
| - **45-64** |  |  | 0.50[0.49-0.51] |  | 0.20[0.20-0.21] |  | 0.46[0.46-0.46] |  | 0.17[0.17-0.18] |  |
| - **65-74** |  |  | 0.49[0.48-0.50] |  | 0.20[0.19-0.21] |  | 0.47[0.46-0.48] |  | 0.18[0.18-0.19] |  |
| - **75-84** |  |  | 0.49[0.46-0.51] |  | 0.22[0.20-0.25] |  | 0.44[0.42-0.47] |  | 0.17[0.15-0.20] |  |
| - **≥85** |  |  | 0.44[0.39-0.50] |  | 0.16[0.12-0.22] |  | 0.47[0.37-0.58] |  | 0.13[0.07-0.22] |  |
| **<45** | **Men** |  | 0.51[0.49-0.53] |  | 0.20[0.19-0.23] |  | 0.47[0.46-0.47] |  | 0.18[0.17-0.18] |  |
|  | **Women** |  | 0.46[0.44-0.49] |  | 0.17[0.15-0.19] |  | 0.48[0.47-0.48] |  | 0.20[0.19-0.20] |  |
| **45-64** | **Men** |  | 0.51[0.50-0.51] |  | 0.21[0.20-0.22] |  | 0.46[0.45-0.46] |  | 0.17[0.16-0.17] |  |
|  | **Women** |  | 0.48[0.47-0.50] |  | 0.19[0.18-0.20] |  | 0.46[0.46-0.47] |  | 0.18[0.17-0.18] |  |
| **65-74** | **Men** |  | 0.49[0.48-0.50] |  | 0.21[0.20-0.22] |  | 0.46[0.44-0.47] |  | 0.18[0.17-0.19] |  |
|  | **Women** |  | 0.50[0.48-0.51] |  | 0.19[0.17-0.21] |  | 0.48[0.47-0.50] |  | 0.19[0.18-0.20] |  |
| **75-84** | **Men** |  | 0.48[0.45-0.51] |  | 0.23[0.20-0.27] |  | 0.44[0.40-0.48] |  | 0.17[0.13-0.21] |  |
|  | **Women** |  | 0.49[0.46-0.53] |  | 0.21[0.18-0.25] |  | 0.44[0.41-0.48] |  | 0.18[0.15-0.21] |  |
| **≥85** | **Men** |  | 0.46[0.38-0.56] |  | 0.14[0.09-0.22] |  | 0.52[0.35-0.78] |  | 0.10[0.04-0.29] |  |
|  | **Women** |  | 0.43[0.36-0.51] |  | 0.18[0.12-0.27] |  | 0.44[0.33-0.58] |  | 0.14[0.07-0.27] |  |

* adjusted for age and 2017-2019 time trends; W: weeks

e-Table 4: Number of **people having at least one** lipid blood profiling or Holter ECG and the number of **overall** lipid blood profiling and Holer ECG from 2017 to 2021

|  |  | **Lipid blood profiling** | | | | | | | | |  | **Holter ECG** | | | | | | | | |  |
| --- | --- | --- | --- | --- | --- | --- | --- | --- | --- | --- | --- | --- | --- | --- | --- | --- | --- | --- | --- | --- | --- |
|  |  | **2017** |  | **2018** |  | **2019** |  | **2020** |  | **2021*** |  | **2017** |  | **2018** |  | **2019** |  | **2020** |  | **2021*** |  |
| **Numbers of persons who had at least one reimbursement** | | | | | | | | | | | | | | | | | | | | | |
| **All the year** |  | 21223099 |  | 21503840 |  | 22132477 |  | 21241386 |  | NA |  | 832085 |  | 867344 |  | 894856 |  | 906514 |  | NA |  |
| **Numbers of reimbursements (the same person might have several lipid blood profiles or Holter ECGs)** | | | | | | | | | | | | | | | | | | | | | |
| •        **January** |  | 2232705 |  | 2488179 |  | 2461680 |  | 2628380 |  | 2641541 |  | 86468 |  | 89760 |  | 92758 |  | 105168 |  | 100691 |  |
| •        **February** |  | 2310781 |  | 2239266 |  | 2373536 |  | 2562465 |  | 2569623 |  | 78762 |  | 82676 |  | 85156 |  | 95993 |  | 96493 |  |
| •        **March** |  | 2762020 |  | 2712441 |  | 2749300 |  | 1616973 |  | 3002710 |  | 94747 |  | 92303 |  | 92389 |  | 64226 |  | 114814 |  |
| •        **April** |  | 2201502 |  | 2332278 |  | 2457436 |  | 1140000 |  | 2655094 |  | 73290 |  | 83516 |  | 88598 |  | 37641 |  | 100986 |  |
| •        **May** |  | 2328766 |  | 2284259 |  | 2478710 |  | 2227055 |  | *1804735* |  | 81850 |  | 79397 |  | 87319 |  | 72156 |  | *67309* |  |
| •        **June** |  | 2327196 |  | 2471572 |  | 2318800 |  | 2923742 |  |  |  | 85416 |  | 91655 |  | 84284 |  | 103770 |  |  |  |
| •        **July** |  | 1889334 |  | 1894168 |  | 2078004 |  | 2374021 |  |  |  | 67403 |  | 77814 |  | 83309 |  | 93344 |  |  |  |
| •        **August** |  | 1809259 |  | 1757839 |  | 1864432 |  | 1998896 |  |  |  | 55624 |  | 56769 |  | 56180 |  | 66906 |  |  |  |
| •        **September** |  | 2597424 |  | 2470920 |  | 2546942 |  | 2679728 |  |  |  | 82312 |  | 81297 |  | 88286 |  | 101831 |  |  |  |
| •        **October** |  | 2555831 |  | 2631093 |  | 2672233 |  | 2669460 |  |  |  | 87669 |  | 95160 |  | 96921 |  | 100759 |  |  |  |
| •        **November** |  | 2418042 |  | 2443636 |  | 2504294 |  | 2451715 |  |  |  | 87242 |  | 90415 |  | 87515 |  | 99112 |  |  |  |
| •        **December** |  | 2071463 |  | 2088954 |  | 2169502 |  | 2396983 |  |  |  | 73416 |  | 73913 |  | 81374 |  | 92531 |  |  |  |
| **Entire year** |  | 27504323 |  | 27814605 |  | 28674869 |  | 27669418 |  | NA |  | 954199 |  | 994675 |  | 1024089 |  | 1033437 |  | NA |  |

* Up to week 20 (23 May 2021); ^b^rates standardized based on the age structure of the 2017 French census population; ^c^rates per 100,000 inhabitants.

Italics: month of May incomplete for the year 2021. NA: not applicable

e-Figure 1: Incidence rate ratio (IRR)^a^ and 95% confidence intervals (95% CI) between rates of initiation of treatments respectively in 2020 and 2021 compared to the rates of initiation in 2017-2019 **according age group**

| **Initiation** | **IRRs 2020 versus 2017-2019** | **IRRs 2021 versus 2017-2019** |
| --- | --- | --- |
| **Antihypertensive medication** |  |  |
| **Lipid-lowering medication** |  |  |
| **Oral anticoagulants in atrial fibrillation indication** |  |  |
| **Prescribed smoking cessation medications** |  |  |

^a^adjusted for age and 2017-2019 time trends; IRRs: Incidence rate ratios; Blue: men; Orange: women.

*p-value for sex-interaction <0.05.

e-Figure 2: Incidence rate ratio (IRR)^a^ between the treatment initiation in 2020 and 2021 respectively versus 2017-2019 **according to previous history of cardiovascular diseases (CVD).**

1. Initiation of antihypertensive medication

*****

*****

*****

*****

*****

*****

*****

*****

*****

*****

*****

*****

*****

*****

*****


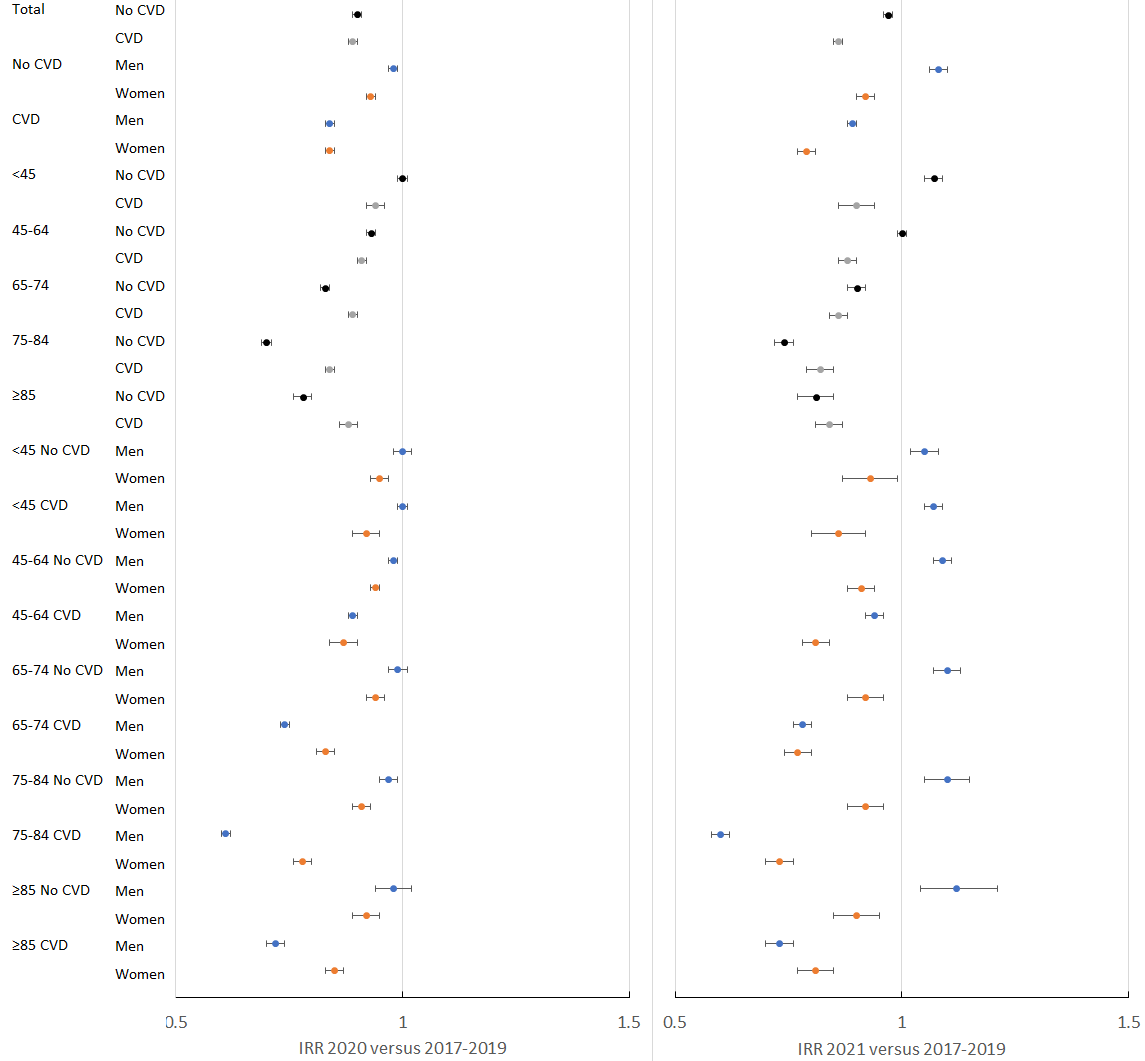


*****

*****

*****

*****

*****

*****

*****

*****

*****

*****

*****

1. Initiation of lipid-lowering medication


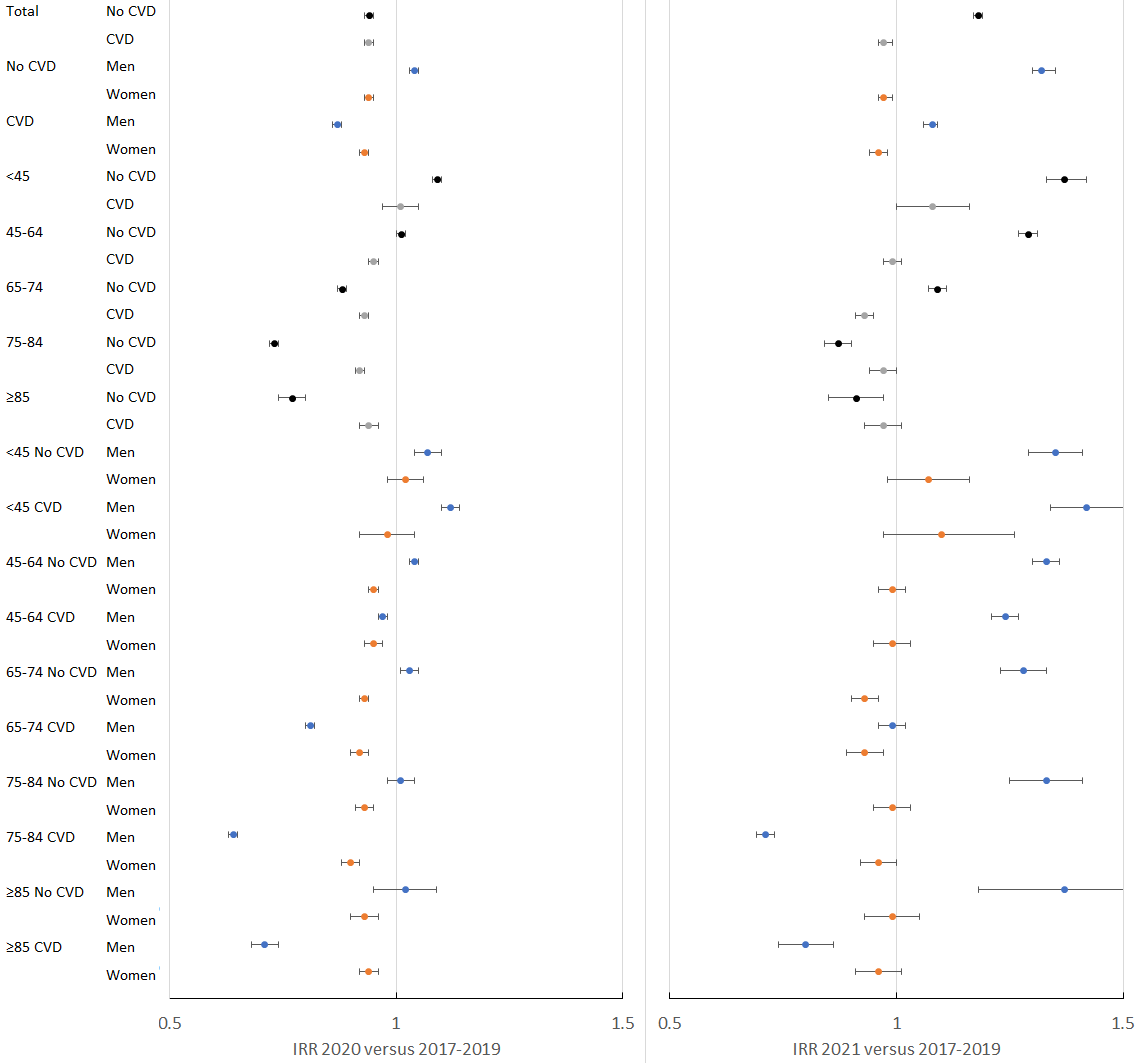


*****

*****

*****

*****

*****

*****

*****

*****

*****

*****

*****

*****

*****

*****

*****

*****

*****

*****

*****

*****

*****

*****

*****

*****

*****

*****

*****

*****

*****

*****

1. Initiation of prescribed smoking cessation medications

*****

*****

*****

*****


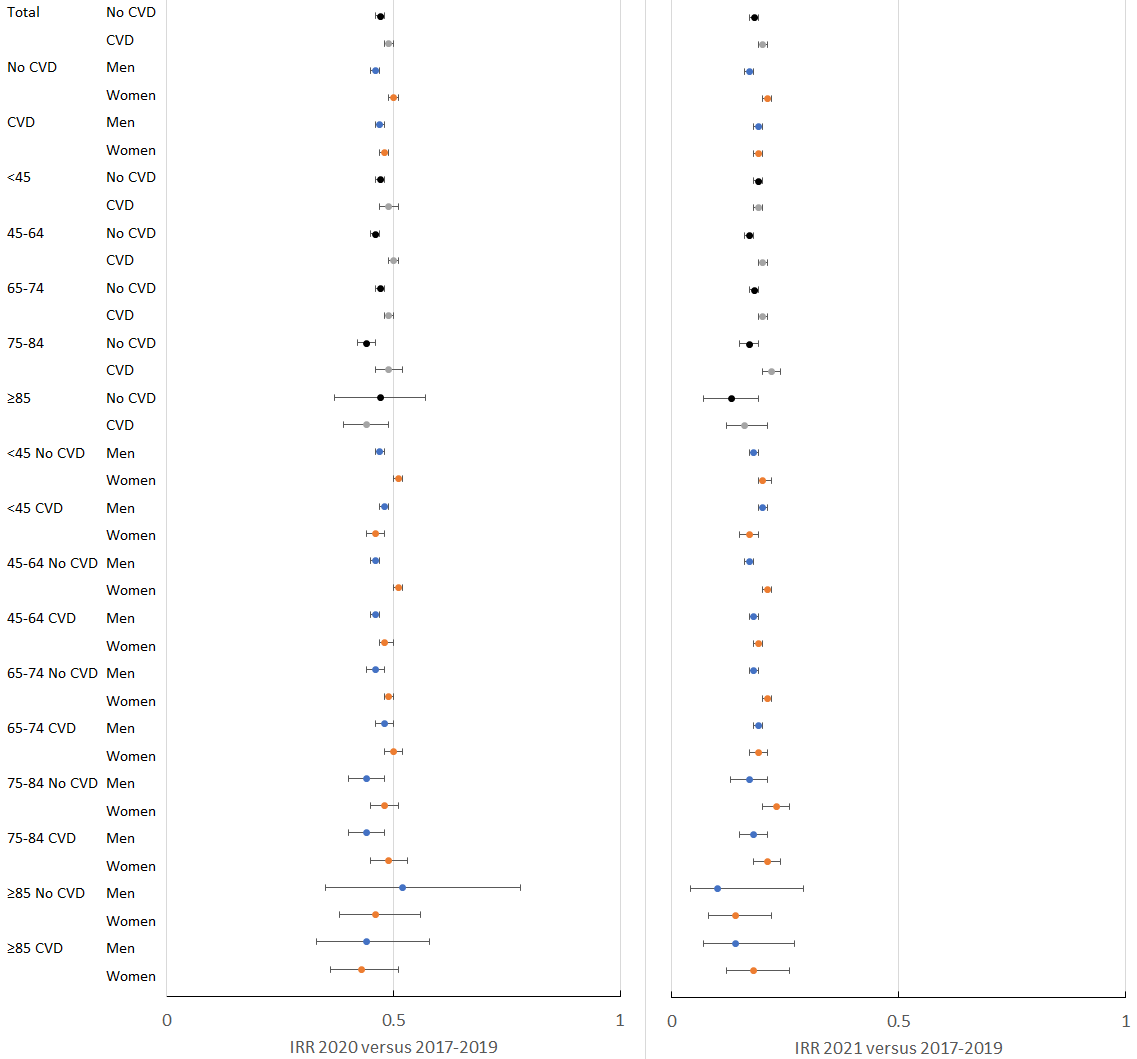


*****

*****

*****

*****

^a^adjusted for age and 2017-2019 time trends; W: weeks; Black: no history of cardiovascular disease (CVD); Gray: history of CVD; Blue: men; Orange: women.

*p-value for sex-interaction or history of CVD-interaction <0.05.

# Sensitivity analysis

We conducted a sensitivity analysis using an interrupted time series analysis (ITS) to evaluate level changes in the initiation of medication of interest introduced by the COVID-19 pandemic. The data were combined by week between January 2017 and the 23 of May 2021 (week 20). Three ruptures were defined: the first one, on the 9 of March 2020 (week 11/impact 1) – start of overwhelmed hospitals and two weeks before the first national lockdown-, the second one on the 11 of May 2020 (week 20/ impact 2) starting at the end of the first lockdown, a third one on the 26 of October 2020 (week 44/ impact 3) indicating the beginning of the second national lockdown which was followed by a curfew and a 3^rd^ national lockdown. We modelized the number of patients who initiated a treatment of interest as time series using a Generalized Additive Model with a Poisson distribution and a log link function (Bernal et al. 2016, Bhaskaran et al 2013, Woods et al 2017)(1-3). The log of population census was used as offset. Seasonality was taking into account using penalized cubic regression splines (Woods et al (Wood S. N. (2017. a)), Bhaskaran et al 2013). The penalty was estimated using Generalized Cross Validation (GCV) criteria. We introduced a dummy variable to take into account the decrease in initiation during Christmas and new year’s weeks. Residual autocorrelation was checked by visualizing autocorrelation and the partial autocorrelation function. No residual autocorrelation was found after taking into account seasonality. We checked for overdispersion which was not found in our study. The final model can be resumed as followed:

Log(N)= Β_0_ + β_1_*time_t_

+ β_2_*impact1_t_ + β_3_*time after impact1_t_

+ β_4_*impact2_t_ + β_5_*time after impact2_t_

+ β_6_*impact3_t_ + β_7_*time after impact3_t_

+ s(week) + s(age) + new_year_week + christmas_week + e_t_

Where: N was the number of patients initiating a treatment of interest, s() penalized cubic regression splines, and the logarithm of the population census was used as offset parameter. Time after impact was centered at the middle of the time window so that the coefficients can be interpreted as mean impact during each time window.

We stratified analyses by gender and age groups. Results were presented below in supplementary e-Table 5 through incidence rate ratio (IRR) calculated as the exponential of the parameter estimates of the ITS analysis and corresponding percent changes with 95% confidence interval calculated as followed: (IRR-1)*100. For information we also provided rates of initiation predicted by the ITS analysis with and without the impact of COVID-19 (see below e-Figure3).

We found the same results than in our main analysis regarding level changes. Our first approach was conserved because we chose an easier method to control for seasonality but also because the ITS analysis seemed not appropriate to analyze changes that occurred since the COVID-19 pandemic as the ITS model considered them as linear changes.

e-Table 5: Incidence rate ratio (IRR), corresponding percent change and 95% confidence intervals (95%CI) between the rates of treatment initiation before the COVID-19 pandemic compared to the rates of initiation after the COVID-19 pandemic (level change) according a sensitivity analysis using an interrupted time series modelling

|  |  | **Antihypertensive medication** | |  | **Lipid-lowering medication** | |  | **Oral anticoagulants in atrial fibrillation indication** | |  | **Prescribed smoking cessation medications** | |  |
| --- | --- | --- | --- | --- | --- | --- | --- | --- | --- | --- | --- | --- | --- |
| Level Change |  | IRR[95%CI] | Percent change [95%CI]^a^ |  | IRR[95%CI] | Percent change [95%CI] ^a^ |  | IRR[95%CI] | Percent change [95%CI] ^a^ |  | IRR[CI95%] | Percent change [95%CI] ^a^ |  |
| All periods combined |  | 0.91[0.91-0.91] | -9.2[-9.5;-9.0] |  | 0.95[0.95;0.96] | -4.5[-4.9;-4.2] |  | 0.95[0.94;0.95] | -5.1[-5.7;-4.5] |  | 0.49[0.48;0.49] | -51.3[-51.5;-51.1] |  |
| Impact 1 |  | 0.67[0.66;0.67] | -33.5[-34.1;-32.9] |  | 0.54[0.54;0.55] | -45.7[-46.5;-45] |  | 0.63[0.61;0.64] | -37.4[-38.6;-36.1] |  | 0.41[0.41;0.41] | -58.9[-59.4;-58.5] |  |
| Impact 2 |  | 1.15[1.14;1.16] | 14.7[13.7;15.8] |  | 1.29[1.27;1.30] | 28.8[27.2;30.5] |  | 1.12[1.09;1.14] | 11.6[9.5;13.7] |  | 1.02[1.01;1.03] | 2.0[1.1;3.0] |  |
| Impact 3 |  | 0.99[0.98;0.99] | -1.2[-1.7;-0.6] |  | 1.00[0.99;1.00] | -0.2[-1.0;0.5] |  | 0.95[0.94;0.96] | -5.2[-6.3;-4.1] |  | 0.99[0.98;1.00] | -1.1[-1.9;-0.4] |  |
| Men |  | 0.97[0.96-0.97] | -3.5[-3.9;-3.1] |  | 0.99[0.99;1.00] | -0.6[-1.2;-0.1] |  | 0.98[0.97;0.98] | -2.3[-3.1;-1.5] |  | 0.49[0.49;0.49] | -51.0[-51.3;-50.7] |  |
| Women |  | 0.87[0.86-0.87] | -13.4[-13.7;-13.1] |  | 0.92[0.91;0.92] | -8.2[-8.6;-7.7] |  | 0.92[0.91;0.93] | -7.9[-8.7;-7.1] |  | 0.48[0.48;0.48] | -51.8[-52.1;-51.5] |  |
| p_interaction_^b^ |  | **<0.0001** | |  | **<0.0001** | |  | **<0.0001** | |  | **<0.0001** | |  |
| <45 |  |  |  |  |  |  |  |  |  |  |  |  |  |
| Men |  | 0.97[0.96;0.98] | -3.1[-4.0;-2.1] |  | 1.06[1.04;1.08] | 6.0[4.2;7.8] |  | 0.95[0.92;0.99] | -4.5[-7.7;-1.2] |  | 0.50[0.49;0.50] | -50.5[-50.9;-50.0] |  |
| Women |  | 0.99[0.98;1.00] | -1.1[-1.9;-0.4] |  | 1.08[1.06;1.10] | 8.2[5.9;10.5] |  | 0.96[0.93;1.00] | -3.7[-7.0;-0.4] |  | 0.51[0.50;0.51] | -49.2[-49.7;-48.8] |  |
| p_interaction_^b^ |  | **<0.0001** | |  | 0.3180 | |  | **0.9607** | |  | **<0.0001** | |  |
| 45-64 |  |  |  |  |  |  |  |  |  |  |  |  |  |
| Men |  | 0.96[0.96;0.97] | -3.8[-4.3;-3.2] |  | 1.01[1.00;1.02] | 0.8[0.0;1.5] |  | 0.99[0.97;1.00] | -1.5[-3.1;0.2] |  | 0.50[0.49;0.50] | -50.5[-50.9;-50.1] |  |
| Women |  | 0.89[0.89;0.90] | -10.7[-11.2;-10.2] |  | 0.98[0.97;0.99] | -2.1[-2.9;-1.3] |  | 0.93[0.91;0.95] | -7.0[-9.0;-5.1] |  | 0.47[0.47;0.48] | -52.8[-53.2;-52.3] |  |
| p_interaction_^b^ |  | **<0.0001** | |  | **<0.0001** | |  | **<0.0001** | |  | **0.0066** | |  |
| 65-74 |  |  |  |  |  |  |  |  |  |  |  |  |  |
| Men |  | 0.98[0.97;0.99] | -1.8[-2.7;-0.9] |  | 0.98[0.97;0.99] | -1.8[-2.8;-0.8] |  | 0.98[0.96;0.99] | -2.1[-3.6;-0.6] |  | 0.50[0.49;0.51] | -49.6[-50.5;-48.6] |  |
| Women |  | 0.79[0.78;0.80] | -20.9[-21.5;-20.2] |  | 0.89[0.88;0.89] | -11.4[-12.2;-10.6] |  | 0.91[0.89;0.92] | -9.3[-11.0;-7.7] |  | 0.51[0.50;0.52] | -49.4[-50.4;-48.3] |  |
| p_interaction_^b^ |  | **<0.0001** | |  | **<0.0001** | |  | **<0.0001** | |  | **0.3373** | |  |
| 75-84 |  |  |  |  |  |  |  |  |  |  |  |  |  |
| Men |  | 0.96[0.95;0.98] | -3.7[-5.0;-2.4] |  | 0.96[0.94;0.97] | -4.4[-5.7;-3] |  | 0.96[0.94;0.97] | -4.2[-5.7;-2.6] |  | 0.49[0.47;0.51] | -51.0[-53.3;-48.6] |  |
| Women |  | 0.72[0.71;0.73] | -27.8[-28.6;-27.1] |  | 0.81[0.80;0.82] | -19.0[-20.0;-18.0] |  | 0.89[0.87;0.90] | -11.2[-12.6;-9.8] |  | 0.50[0.48;0.53] | -49.9[-52.3;-47.4] |  |
| p_interaction_^b^ |  | **<0.0001** | |  | **<0.0001** | |  | **<0.0001** | |  | **0.7076** | |  |
| ≥85 |  |  |  |  |  |  |  |  |  |  |  |  |  |
| Men |  | 0.97[0.96;1.00] | -2.5[-4.5;-0.5] |  | 0.94[0.91;0.96] | -6.1[-8.5;-3.6] |  | 0.95[0.93;0.97] | -5.4[-7.4;-3.3] |  | 0.74[0.62;0.87] | -26.4[-37.8;-13.0] |  |
| Women |  | 0.83[0.82;0.84] | -17.2[-18.3;-16.0] |  | 0.89[0.87;0.91] | -11.2[-12.8;-9.5] |  | 0.91[0.90;0.92] | -9.0[-10.5;-7.6] |  | 0.69[0.60;0.80] | -31.1[-40.4;-20.4] |  |
| p_interaction_^b^ |  | **<0.0001** | |  | **0.0380** | |  | **0.0441** | |  | **0.1932** | |  |

^a^(IRR-1)*100; ^b^p-value for the interaction between men and women; bold: p-value for interaction <0.05.

e-Figure 3: Weekly rates of initiation per 1000 inhabitants according to time series analysis, with observed rates (gray points), predicted rates with COVID-19 pandemic (red curve) and predicted rates without the COVID-19 pandemic (black curve)

1. Antihypertensive medications


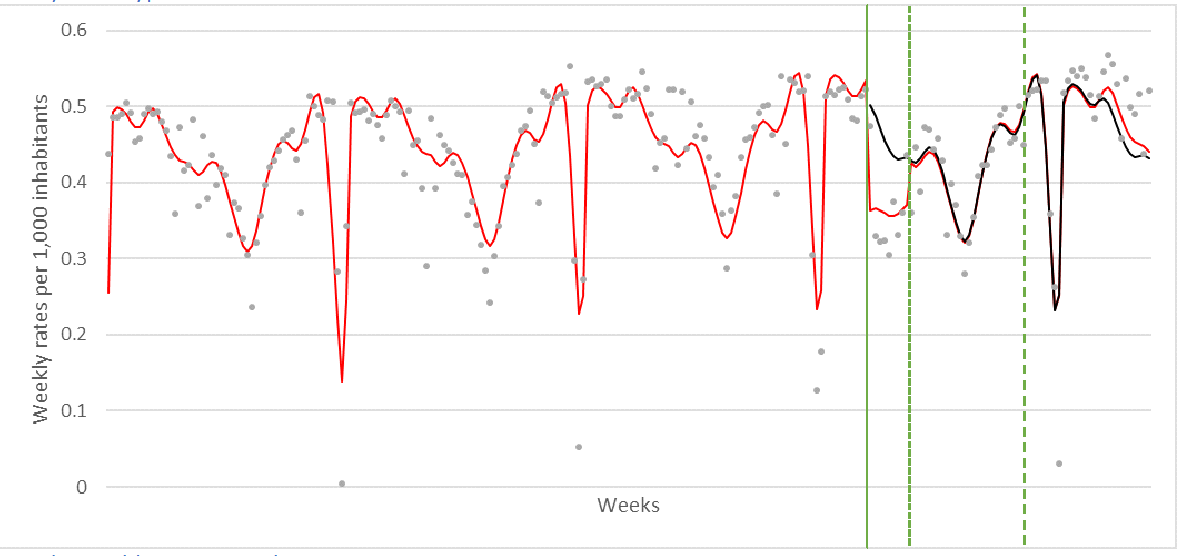


1. Lipid-lowering medications


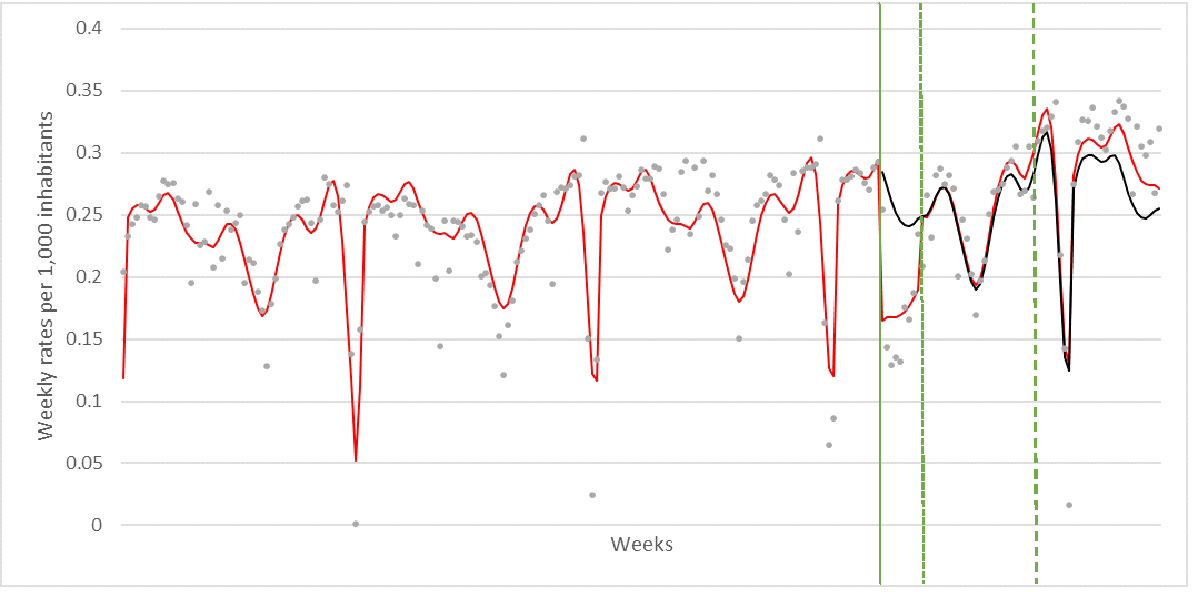


1. Oral anticoagulants in atrial fibrillation indication


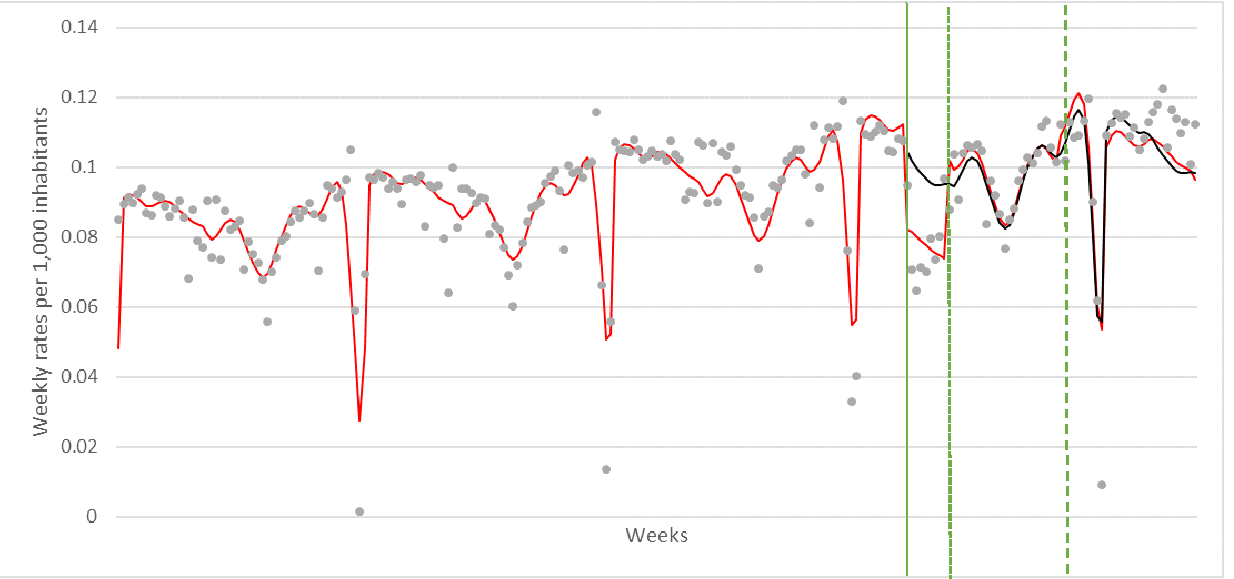


1. Prescribed smoking cessation medications


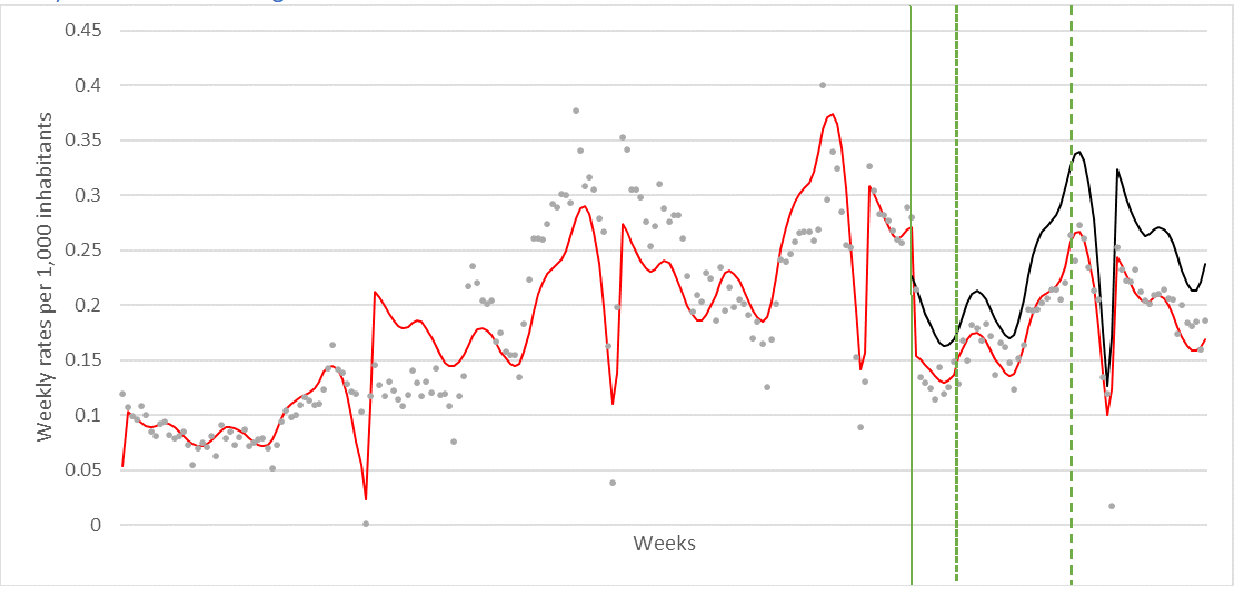


Legends:

- gray points = observed rates
- red lines = ITS prediction including the COVID-19 pandemic
- black lines = prediction excluding the COVID-19 pandemic
- green lines = Three ruptures defined in the ITS model the 9 of March 2020 (week 11/impact 1), the 11 of May 2020 (week 20/ impact 2), and the 26 of October 2020 (week 44/ impact 3)

1. Bernal JL, Cummins S, Gasparrini A. Interrupted time series regression for the evaluation of public health interventions: a tutorial. Int J Epidemiol. 2017;46(1):348-55.

2. Bhaskaran K, Gasparrini A, Hajat S, Smeeth L, Armstrong B. Time series regression studies in environmental epidemiology. Int J Epidemiol. 2013;42(4):1187-95.

3. Wood SN. Generalized Additive Models: An Introduction with R: CRC Press/Taylor & Francis Group; 2017.
